# The Five-Dimension Patient Experience Model and Its Applications in Healthcare Using the Example of Spinal Cord Injury and COPD

**DOI:** 10.1177/23743735241293965

**Published:** 2024-10-30

**Authors:** Carlos Bezos Daleske

**Affiliations:** 1 661302 Instituto para la Experiencia del Paciente (IEXP), Majadahonda, Spain

**Keywords:** patient engagement, patient expectations, patient experience model, patient experience conceptual model, patient experience applications, dimensions of patient experience

## Abstract

The need for a comprehensive patient experience model across healthcare sectors is crucial. This study introduces IEXP's five-dimension model: physical, emotional, social, cultural, and healthcare. Unlike hospital-centric frameworks, this model acknowledges holistic experiences, vital for chronic conditions. Applied to chronic obstructive pulmonary disease (COPD) and spinal cord injury (SCI), it showcases its adaptability. This model benefits healthcare, patient groups, pharmaceuticals, and medical tech, optimizing care, policy support, adherence, and patient-focused tech. Despite limitations and a chronic condition focus, the model adds value in shaping patient experience cross-sector.

## Introduction

As health organizations tend to focus on patient experience^1^—for instance as an indicator of performance^2^ or because of its impact in clinical outcomes or efficiency^3-5^ there is a need for a comprehensive conceptual patient experience model, that can be applied in a practical way by healthcare providers.

There are already some frameworks in patient experience that can be traced back to the Donabedian quality model. This model focuses on the structure, process, and outcomes of medical care.^6,7^ A reference framework is the NHS Patient Experience Book.^8^ Likewise Birkelien^9^ has developed a comprehensive framework based on seven points going from cultures up to quality outcome maximization, passing through hospital environment or engagement.

All these frameworks and models introduce important perspectives both from the conceptual and the practical side. Yet most of them have been developed for the experience at hospitals. But the patient experience goes beyond hospital care, especially in the case of people with chronic diseases. Most of their experience happens outside the hospital and disease management consists mainly in self-management.^10,11^ At this point we must also consider the family and social context of people with illnesses, which is part of the experience both with the pathology and with care.

The Institute of Patient Experience (IEXP) has developed a model of patient experience based on five dimensions which encompasses an integrated approach of all the experience and that can be applied in practice also outside hospitals; for instance, in home care or disease self-management.

This article will, first, describe the conceptual background from which the five dimensions of the patient experience emerge. Second, it will analyze the patient experience also outside of the hospital using two chronic conditions as an example in order to explore the dimensions that can be found according to the conceptual framework. Third it will describe some practical applications of the model before the discussion section, where findings will be compared against literature.

## Methodology

In order to explore the dimensions patient experience is made of, this article will analyze patient experience in chronic diseases. From the many possible chronic conditions that could be analyzed, chronic obstructive pulmonary disease (COPD) and spinal cord injury (SCI) were chosen for their distinct patterns of healthcare utilization—characterized by periods of intensive medical interventions and extended out-of-hospital care—and the significant involvement of family, caregivers, and social services.

The source of the insights and data on both conditions come from a European Union (EU) funded project for remote rehabilitation the author participates at, so direct observation, as well as interviews with actors, have been possible. The project called Rosia aims to set up digital remote rehabilitation for seven diseases, COPD and SCI among them. One of the reasons to choose this particular project is precisely its digital approach that can highlight the application of the model to this framework. As an EU funded project results are open and can be used in this research.

Rosia^12^ is an EU funded project that aims to develop remote self-care and rehabilitation in isolated areas for seven conditions: cardiovascular disease, COPD, acquired brain injury, SCI, knee arthroplasty, COVID-19 (long covid), and hip fracture. A comprehensive patient experience analysis was conducted prior to specifying the requirements for procurers in order to redesign rehabilitation services to better align with patients’ realities, needs, and expectations.

The patient experience research was done using semi-structured interviews with healthcare professionals and online workshops both with patients and professionals. The semi-structured interviews were meant to map the patient flow. The workshops were designed to gain qualitative insights of the patient experience in hospital and at home.

Finally, the article will briefly describe three practical applications of the five-dimension patient experience model to improve patient experience in chronic diseases.

## Conceptual Framework of the Five Element Patient Experience Model

According to the World Health Organization, health defined as “a state of complete physical, mental and social well-being and not merely the absence of disease or infirmity.”^13^ De la Guardia et al undertake a close analysis of the definition underlying the mental and social dimensions.^14^ In fact, much attention has been paid by literature to the clinical impact of emotions^15-19^ and about the importance of social determinants of health.^14,20^ More recent research has found out, that culture can also act as a determinant of health^21,22-27^ as the rich tradition of medical and clinical anthropology shows.^28-32^ There is also research about the impact of cultural believes and therapeutic adherence.^33-36^

Finally, the Beryl Institute defines patient experience as “the sum of all interactions, shaped by an organization's culture, that influence patient perceptions across the continuum of care.”^37^ This definition can be enlarged by Wolf's et al approach stressing the importance of the continuum of care provided by organizations and looking beyond surveys by focusing on expectations and on personalized care.^38^

Thus, the framework to develop the model is based on the WHO health definition that sees health not just as the absence of disease, but as physical, mental, and social well-being. Although the model is based on the WHO definition, it strongly emphasizes the importance of the care, emotional, social, and cultural factors that have been briefly described above.

Emotions play a significant role in the patient experience, impacting health outcomes, and requiring strategies for emotional well-being. Social determinants of health, such as socioeconomic status, education, and social support networks, influence a patient's well-being and should be addressed. Additionally, cultural determinants, including beliefs, values, and behaviors,^21,22,26,33,39-42^ shape the patient experience and require culturally sensitive and competent care. And finally, the healthcare system is the physical, institutional, and social field,^43,44^ were all these experiences take place. But not only, in the case of chronic diseases, accidents and other events, the physical, institutional, and social field of the conditions can be extended to home, family, workplace, or community.

Based on the framework, the patient model has five dimensions: physical, healthcare, emotional, social, and symbolic (cultural). The physical aspect focuses on treatment effectiveness or pain management. Healthcare involves interactions with providers, including communication, timeliness, and patient involvement. Psychological and emotional aspects are part of the emotional dimension. The social dimension includes impacts like stigma, categorization, and work consequences, considering support networks, resources, and engagement. Cultural beliefs play a role in the symbolic dimension, involving competency, respect, and care.

## Analysis of Patient Experience in COPD and SCI and Exploration of Existing Dimensions

These five dimensions can be observed using the patient experience analysis done for the Rosia Project.

### COPD Patient Experience: Insights From Rehabilitation

In the ROSIA project, COPD rehabilitation patients displayed reduced awareness of COPD's impact, leading to partial adherence to medication and rehab regimens. Medication preference over comprehensive interventions persisted, despite overall satisfaction. Travel burden and distrust of telerehabilitation due to tech insecurities were common challenges.

COPD patients’ families adjusted social roles and routines, experiencing varied emotions. Guilt from smoking's self-inflicted nature prevailed. Dependency and depression arose from limitations and family changes. Motivation emerged with rehabilitation progress. Families felt conflicting emotions while supporting patients.

Rehabilitation acknowledges the incurable condition but enhances life quality. Patient responses vary from anger to motivation, tied to personal histories and guilt from smoking. Motivation counters resignation and depression.

Clinicians drive telerehabilitation success via monitored progress, family support, and counseling. Visible progress enhances life quality, aiding reintegration. Follow-ups and online training boost adherence. Institution success involves collaboration, empowerment, and psychological support, transforming COPD experience.

### Patient Experience Dimensions in COPD

Travel discomfort and doubts about telerehabilitation's viability reveal an underlying physical dimension beyond COPD's overt symptoms. This extends beyond visible coughing and fatigue, highlighting mobility's role. Patients suffer often of depression for many reasons; one is the fact that the condition is not curable and another other is that the lack of mobility seriously limits social interactions with family and other social actors.

Emotions like guilt, dependency, and fleeting depression offer a window into COPD patients’ emotional dimension, mirroring psychological challenges.

Adaptations by families and caregivers highlight a social dimension in COPD. These shifts reveal patient interconnectedness, reflecting broader societal influence on social dynamics.

Guilt's emotional response, tied to self-inflicted COPD through smoking, reflects a cultural or symbolic layer in patient encounters. This sentiment embodies a cultural dimension influencing perception and response to the condition.

### Experience of Spinal Cord Injury Patients With Rehabilitation

Post-SCI, a weekly rehabilitation rhythm emerges, causing anxieties and dependency. Patients struggle with basic tasks, feeling burdensome. This interplay of physical, psychological, emotional spheres can lead to depression, impacting recovery.

SCI patients see it as a life-altering point, adapting to new norms. Initial rehab brings challenges—eating, going out require adaptation. SCI experience centers on transformation—surviving shock, embracing new reality, and creating a better life. Obstacles exist—long hospital waitlists, limited community resources hamper post-discharge rehab. Physical, psychological, and social adaptations are needed.

### Patient Experience Dimensions in SCI

Discussions on physiotherapists, challenges with healthcare professionals, and “long waiting lists” reveal healthcare's crucial role and systemic barriers. Emotional nuances emerge, referencing “guilt,” “anxiety,” and “depression,” highlighting SCI's emotional impact. “Dependence” and “self-esteem loss” echo their emotional journey.

Research implies the social dimension through family and caregiver adjustments, indicating broader SCI impacts. “Peers and patient associations” signify social networks’ role.

Cultural undercurrents emerge, seen in “guilt” and adapting to new family roles. “Lack of knowledge about SCI” reflects culture's influence on perceptions and information dissemination.

Patient experience analysis within COPD and SCI rehabilitation, from workshop insights, reveals interwoven sub-experiences in journey dimensions: physical, healthcare, emotional, social, and cultural. Physical aspect, seen in mobility issues, medication preference, and emotional reactions, links to healthcare choices, reflecting medical options’ well-being interplay. This extends to social context, evident in familial adaptations, underlining broader societal influence. Cultural dimension's impact reflects attitudes, symbolic layers shaping patient encounters.

## Practical Applications

The described conceptual model of five dimensions has practical applications in the improvement of the patient experience and disease management in chronic conditions beyond hospital care. Three still ongoing IEXP projects will be described: improvement of the patient experience and processes in dermatology; use of patient experience to ensure better derivations to reference centers in sarcoma; digital care for remote rehabilitation.

### Improving Experience and Processes in Dermatology

ExpaDerm is a project designed to improve healthcare quality for atopic dermatitis and psoriasis in dermatology departments. By the time this article was written it has been deployed in 14 Spanish hospitals since 2022 and it is still going on. Patients benefit from psychological support, earlier referral, treatment of outbreaks between consultations, empowerment actions for disease self-management, social awareness against stigma, and so on Hospitals benefit from less frequentation, better adherence, and less use of resources and also from smoother protocols and processes.

The project uses a participatory action research approach were patients and healthcare professionals reach a common diagnosis, identify barriers and opportunities, and develop jointly solutions. Including the five dimensions helped participants to address not only their main pains, namely the psychological burden and a faster treatment of outbreaks, but to understand that involving families and working with other patients (social dimension) means a better quality of life, psychological relieve and better disease management. Introducing reflections on the cultural patterns on stigma is essential in order to design personal and collective strategies against stigma.

### Derivations in Sarcoma

The five-dimension patient experience model was used to produce evidence that could help to produce better protocols on derivation in sarcoma. The Spanish law provides a series of national reference centers where hospitals have to refer sarcoma patients. Nevertheless, many hospitals do not do so for a series of reasons going from ignorance of the law to political ones.

Together with Asarga, a Spanish sarcoma patient organization, IEXP started a study on the sarcoma patient experience comparing patients that were referred to a specialized center with those that were not referred. The study included in its literature review plenty of previous evidence of the therapeutic an economic benefit of early derivation in UK and France. The still unpublished qualitative study showed that survival and quality of life of referred patients were higher than in those of not referred patients, mainly to better and quicker diagnosis and treatment. Participants highlighted the importance of considering the sarcoma patient care process as an emotional and psychological experience, with the need to provide multidisciplinary care and to improve access to information and appropriate care for patients and caregivers. The experience of patients not referred or referred late was also marked by psychological and economic burden.

Using the five-dimension model, the study addressed the physical and healthcare aspects of survival and quality of life, but also the emotional and psychological impact, the socially neglected caregivers, the economic burden for families and the cultural patterns in many hospitals that prevented them from referral; mainly communication, unclear protocols, preference for local treatment as a way to retain competencies and independence. Thus, the study structured on the five dimensions model helped to transform an organization's claims in evidence that has been used to gain physician support, present the study in the parliament, and start negotiations with political parties as well as with the Ministry of Health. Currently a second project has started to help the Reference Centers in Spain to create shared protocols and use shared resources.

### Digital Care

The study of patient experience for the seven Rosia conditions was the key for identifying patient needs, shortcomings of current rehabilitation processes and all context factors key for the definition of user requirements and the design of use cases. Most context factors are of emotional, social, and cultural nature, thus showing the strength of the model. A patient journey map organized around the five dimensions is an interesting instrument to analyze the current experience with rehabilitation and out from this point design a desired patient experience with rehabilitation technology.

For example, the current emotional experience of SCI patients is defined by a feeling of being left behind by the system, a feeling of dependency and a need to have time to adapt. The rehabilitation technology designer can provide a step by step adaption to the new situation and of support by remote healthcare professionals to counter the feeling of being left behind.

At the end, the five-dimension model provided 19 needs that generated outcomes for technology designers and 27 user requirements that follow into the technology design.

The five-dimensional patient experience model applied in the ROSIA project revealed key insights for remote rehabilitation: the need for adaptive interfaces, continuity of care, addressing emotional isolation, virtual social support, and cultural customization. These insights led to innovations including accessible telemedicine platforms, continuous monitoring systems, and culturally adapted content. Preliminary results indicate improved treatment adherence, reduced travel, and increased patient satisfaction in rural areas, suggesting more effective, personalized rehabilitation solutions for isolated regions.

## Discussion

This article describes the IEXP five-dimension patient experience model and its practical applications in healthcare, using COPD and SCI cases as illustrative examples.

In addition, this article shows its practical applications in healthcare settings, offering insights into enhancing patient care, optimizing rehabilitation strategies, and informing the design of medical technologies to better align with patients’ needs and contexts.

The patient experience not only influences healthcare performance indicators but also impacts clinical results and efficiency as Iwelunmor et al describe in their review of the PEN-3 cultural model and its application in public health research.^23^ Consequently, the development of a comprehensive conceptual patient experience model that can be practically employed across various healthcare contexts has become imperative.

While Donabedian's model and the NHS Patient Experience Book have provided valuable insights, they tend to gravitate toward the hospital-centric sphere.^6-8^ The IEXP model embraces the entirety of patient journeys, extending it to the emotional, social, and cultural context, which is of particular interest in chronic conditions, where self-management and societal factors (mainly family) significantly shape the patient trajectory. By weaving in emotional, social, and cultural dimensions, the IEXP framework contributes to enlarge the continuum of care definition of Wolf et al beyond hospital.^38^

In the realm of COPD rehabilitation, the analysis underscores the interplay of dimensions—physical challenges, healthcare decision-making, emotional responses, social adaptations, and cultural influences—as described by the WHO defining social determinants of health^21^ or analyzed by Soum-Pouyalet et al^40^ in cancer decision-making, Arousell et al in human reproduction.^22^ Likewise, in SCI rehabilitation, the model is able to describe the interconnections from the physical, healthcare, emotional, social, and cultural dimensions.

The current work shows the pragmatic utility of the conceptual IEXP model to improve patient experience and care beyond the hospital. Healthcare providers, as illustrated by the ExpaDerm and sarcoma initiatives, can leverage the model to refine care processes, focusing on psychological support, empowerment, and societal awareness. The Rosia Project's adoption of the model exemplifies its instrumental role in shaping medical technology, infusing emotional, social, and cultural dimensions into design.

## Conclusions

The Five-Dimension Patient Experience Model introduced by IEXP offers a comprehensive framework for understanding patient experiences in healthcare. It encompasses physical, emotional, social, and cultural dimensions. Analyzing chronic conditions like COPD and SCI demonstrates its relevance improving care for chronic diseases. While comprehensive, the model's qualitative nature and focus on chronic conditions are limitations. Nevertheless, it remains a valuable tool for shaping patient-centered care in diverse sectors.

## Limitations

The model's focus on chronic conditions may not encompass the breadth of experiences across acute conditions or disease progression. While the model emphasizes emotional, social, and cultural dimensions, other factors like economics, accessibility, and communication might receive less focus.

The practical cases described here have not yet been published and peer reviewed, since projects are still ongoing. This could affect the relevance and generalizability of the findings. The relatively small sample size in the initial studies may not fully represent the heterogeneity of patient experiences in remote areas. Furthermore, the long-term efficacy and sustainability of the innovations developed based on this model require further longitudinal studies to validate their impact on patient outcomes and healthcare systems.
